# Torsional and Transversal Stiffness of Orthotropic Sandwich Panels

**DOI:** 10.3390/ma13215016

**Published:** 2020-11-06

**Authors:** Tomasz Garbowski, Tomasz Gajewski, Jakub Krzysztof Grabski

**Affiliations:** 1 Institute of Structural Analysis, Poznan University of Technology, Piotrowo Street 5, 60-965 Poznań, Poland; tomasz.garbowski@put.poznan.pl (T.G.); tomasz.gajewski@put.poznan.pl (T.G.); 2 Institute of Applied Mechanics, Poznan University of Technology, Jana Pawła II Street 24, 60-965 Poznań, Poland

**Keywords:** transverse shear stiffness, plate torsion test, corrugated cardboard, sandwich plate, finite element method, shell element

## Abstract

In the present work, an analytical equation describing the plate torsion test taking into account the transverse shear stiffness in sandwich plates is derived and numerically validated. Transverse shear becomes an important component if the analyzed plate or shell is thick with respect to the in-plane dimensions and/or its core has significantly lower stiffness than the outer faces. The popular example of such a sandwich plate is a corrugated cardboard, widely used in the packaging industry. The flat layers of a corrugated board are usually made of thicker (stronger) material than that used for the corrugated layer, the role of which is rather to keep the outer layers at a certain distance, to ensure high bending stiffness of the plate. However, the soft core of such a plate usually has a low transverse shear stiffness, which is often not considered in the plate analysis. Such simplification may lead to significant calculation errors. The paper presents the generalization of the Reissner’s analytical formula, which describes the torsional stiffness of the plate sample including two transverse shear stiffnesses. The paper also presents the implementation of the numerical model of the plate torsion test including the transverse shear stiffnesses. Both approaches are compared with each other on a wide range of material parameters and different aspect ratios of the specimen. It has been proved that both analytical and numerical formulations lead to an identical result. Finally, the performance of presented formulations is compared with other numerical models using commercial implementation of various Reissner–Mindlin shell elements and other analytical formulas from the literature. The comparison shows good agreement of presented theory and numerical implementation with other existing approaches.

## 1. Introduction

Composite materials play an important role in many practical engineering designs. They consist of at least two materials, which are called fiber and matrix material, or are composed of more than one layer. A special class of composite layered materials is sandwich plates; they consist of a soft core and two outer sheets. The internal one supports the faces and provides the overall flexural stiffness of a structure. In composite laminates or sandwich plates and shells, an important role is played by flexural and torsional stiffness. A typical example of such a sandwich plate is the 3-layer corrugated cardboard, in which the core is corrugated and therefore light. The role of the core called “flute” in jargon is to provide shear stiffness while the outer sheets called “liners” ensure the flexural stiffness. Transversal shear effect is especially important in thick plates.

Avilés and coauthors conducted experiments on the torsion of sandwich panels and made measurements of the shear properties [1]. The authors then proposed a shear-corrected model for the prediction of strength of the corrugated sandwich panels [2]. Recently, Garbowski et al. presented a modification of the formula with the coefficients having a physical meaning [3]. The results were compared with the finite element calculations and proved that including the transverse shear effect in the torsion analysis of corrugated boards gave better results.

Over the years, there have been many approaches in the literature for calculations or experimental determination of the transverse stiffness of sandwich and corrugated plates. In 1978, Cohen published a paper on the transverse shear stiffness of laminated anisotropic shells [4]. Nordstrand et al. examined influence of the core shape on the effective transverse shear moduli [5]. The authors compared the results with classical plate theory and finite element method (FEM) calculations. Nordstrand conducted also investigations on post-buckling strength of orthotropic corrugated board panels using the FEM [6]. Shi and Tong proposed an equivalent transverse shear stiffness of the sandwich panels with honeycomb cores [7]. The panels were simply supported and subject to edge compressive loads. In 1997, Nordstrand and Carlsson conducted two tests for measuring the transverse shear stiffness of structural core sandwich plates [8]. They performed the block shear test and three-point bending test and compared the results with FEM and analytical predictions. Altenbach presented a method for determination of the transverse shear stiffness based on the solution of a Sturm–Liouville problem [9]. This approach can be applied both for laminated and/or sandwich plates.

Analytical or numerical analysis of the composites with heterogeneous layers is not trivial. A crucial point in the analysis of such plates, e.g., corrugated cardboards, is a method of simplification of the heterogeneous layer. This process is called a homogenization, a method for obtaining the effective properties of the corrugated core [10,11]. After the homogenization, the core is treated as a homogeneous layer of the composite, which is characterized by the effective properties. These properties should ensure a similar effect in the case of the composite with the homogenized core under external loading like for the composite with the heterogeneous core. In 2003, Hohe proposed a homogenization method based on strain energy for sandwich panels [12]. A crucial assumption in his approach was the equivalence of a representative element of the heterogeneous element and the homogenous element. Buannic et al. used a periodic homogenization method [13]. They obtained the equivalent membrane and pure bending characteristic of periodic plates. Their approach was modified for sandwich panels in order to incorporate the transfer shear effect in the analysis. The method presented by Biancolini [14] was based on a micromechanical representation of the considered plate using the FEM. The stiffness properties were obtained using the energy equivalency between the model and the equivalent plate. Abbès and Guo showed that in order to determine a torsion rigidity of orthotropic sandwich plates, it is possible to decompose the plates into two beams in two directions of the plate [15]. Marek and Garbowski compared two methods of homogenizations based on the classical laminated plate theory and the deformation energy equivalence method [16].

In analytical or numerical analysis of corrugated sandwich panels three approaches are distinguished in the literature: classical laminated plate theory (CLPT), first order shear deformation theory (FOSDT) and higher-order shear deformation theory (HOSDT). Notice that the CLPT do not include the transverse shear effect. The FOSDT has been proposed by Reissner and Mindlin [17,18]. Carlsson et al. used the FOSDT in the analysis of elastic stiffness of corrugated core sandwiches [19]. Hernández-Pérez et al. applied the FOSDT to the sandwich plate twist specimen [20]. They used a Fourier series to obtain the solution and compared the results with the FEM calculations. The approach presented by Hernández-Pérez et al. is limited to soft-core sandwich panels, where the transverse shear effect dominates the elastic effect. Phan and Reddy published a paper on the application of the HOSDT for laminated composite plates [21]. In the literature, one can find also analysis of corrugated sandwich panels using the HOSDT. Analysis of twist behavior of soft-core sandwich panels using HOSDT has been presented by Elmalich and Rabinovitch [22]. They took into account all components of the core stiffness. Their results have shown that the actual stress state differs significantly from that resulting from the CLPT. In the analysis of boxes made from corrugated cardboard, quite a different approach is strength prediction based on empirical formulas [23], including the very popular McKee formula [24], which is very important from a practical point of view.

In this paper, a new generalized form of the Reissner analytical formula describing the torsion of the sandwich plate is proposed. The torsional stiffness and both transverse shear stiffness are taken into account in these considerations. The presented formula is compared with the numerical models and other analytical approaches existing in the literature.

## 2. Materials and Methods

### 2.1. Reissner–Mindlin Plate—Governing Equations

A plate of constant thickness t was under consideration herein. Its middle surface lies in the plane x–y, while the “thickness direction” is related to the *z*-axis. It was assumed that cross sections remain straight during deformation, but they do not necessarily remain normal to the middle surface. The assumption is typical for shear deformable plates.

**Figure 1:**
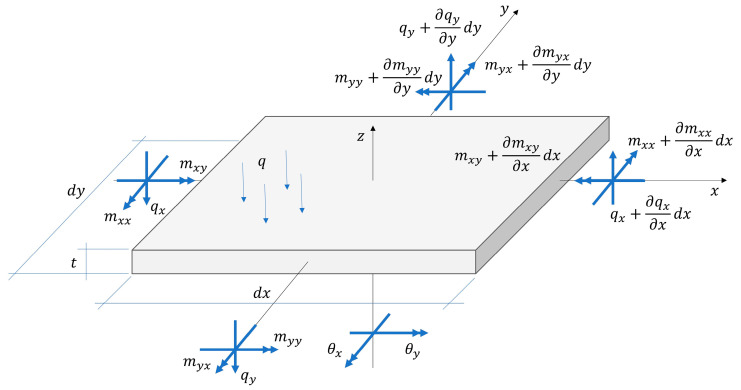
Plate stress resultants and rotations.

The equilibrium equations are expressed in terms of the bending moments $m_{xx}$ and $m_{yy}$, the twisting moments $m_{xy}=m_{yx}$ and the shear forces $q_{x}$ and $q_{y}$. An infinitesimal plate element dA=dx⋅dy subjected to an external transversal load q is depicted in Figure 1, which shows also the stress resultants. The plate equilibrium equations consist of the transversal equilibrium:

(1) $$q_{x,x}+q_{y,y}=q$$

and the rotational equilibrium in x and y:

(2) $$m_{xx,x}+m_{yx,y}-q_{x}=0,$$

(3) $$m_{yy,y}+m_{xy,x}-q_{y}=0,$$

where comma represents the partial derivative of the variable with respect to Cartesian coordinate direction that appears after the comma.

In the present study, w is the transversal displacement and $\theta_{x}$ and $\theta_{y}$ denote the rotations of the plate cross section. Orientation of the rotations is shown in Figure 1. $\gamma_{x}$ and $\gamma_{y}$ denote the shear strains and have the same definitions of the orientations as for the rotations. The kinematic equations take the form:

(4) $$w_{,x}=\theta_{x}-\gamma_{x};\;w_{,y}=\theta_{y}-\gamma_{y}.$$

The curvatures are defined as:

(5) $${𝜅}_{xx}=-\theta_{x,x};\;{𝜅}_{yy}=-\theta_{y,y};$$

(6) $${𝜅}_{xy}={𝜅}_{yx}=-\left(\theta_{x,y}+\theta_{y,x}\right)/2.$$

A linear elastic orthotropic material was assumed here. The plate bending stiffness D and the shear stiffness $D_{s}$ are expressed in terms of the material parameters: $E_{11}$, $E_{22}$, $\nu_{12}$, $\nu_{21}$, $G_{12}$, $G_{13}$, $G_{23}$ and the plate thickness t as:

(7) $$D=\frac{t^{3}}{12}\left[\begin{array}{ccc} \frac{E_{11}}{1-\nu_{12}\nu_{21}} & \frac{\nu_{21}E_{11}}{1-\nu_{12}\nu_{21}} & 0\\ \frac{\nu_{12}E_{22}}{1-\nu_{12}\nu_{21}} & \frac{E_{22}}{1-\nu_{12}\nu_{21}} & 0\\ 0 & 0 & G_{12} \end{array}\right]=\left[\begin{array}{ccc} D_{11} & D_{12} & 0\\ D_{21} & D_{22} & 0\\ 0 & 0 & D_{33} \end{array}\right],$$

(8) $$D_{s}=t\alpha \left[\begin{array}{cc} G_{13} & 0\\ 0 & G_{23} \end{array}\right]=\left[\begin{array}{cc} A_{44} & 0\\ 0 & A_{55} \end{array}\right],$$

where $E_{11}$ is the effective stiffness modulus in the x direction, $E_{22}$ is the effective stiffness modulus in the *y* direction, $\nu_{12}$ is the effective Poisson’s ratio in the 1–2 (xy) plane, $v_{21}=v_{12}E_{22}/E_{11}$, $G_{12}$ is the effective shear modulus in 1–2 (xy) plane, $G_{13}$ is the effective transverse shear modulus in the 1–3 (xz) plane, $G_{23}$ is the effective transverse shear modulus in the 2–3 (yz) plane, $D_{11}$ and $D_{22}$ are bending stiffnesses in two orthogonal directions, $D_{33}$ is the twisting stiffness, $A_{44}$ is the transverse shear stiffness in the 1–3 (xz) plane and $A_{55}$ is the transverse shear stiffness in the 2–3 (yz) plane. The shear correction parameter, α, describes the non-constant distribution of shear stresses through the plate thickness. For a homogenous orthotropic plate with constant thickness, the standard value of α=5/6 was adopted [17,18].

The relation between bending moments and the curvatures can be expressed as:

(9) $$\left\{\begin{array}{c} m_{xx}\\ m_{yy}\\ m_{xy} \end{array}\right\}=\left[\begin{array}{ccc} D_{11} & D_{12} & 0\\ D_{21} & D_{22} & 0\\ 0 & 0 & D_{33} \end{array}\right]\left\{\begin{array}{c} {𝜅}_{xx}\\ {𝜅}_{yy}\\ {𝜅}_{xy} \end{array}\right\},$$

and the shear forces are related to the shear strains by:

(10) $$\left\{\begin{array}{c} q_{x}\\ q_{y} \end{array}\right\}=\left[\begin{array}{cc} A_{44} & 0\\ 0 & A_{55} \end{array}\right]\left\{\begin{array}{c} \gamma_{x}\\ \gamma_{y} \end{array}\right\},$$

where $D_{11}$, $D_{12}=D_{21}$, $D_{22}$, $D_{33}$, $A_{44}$ and $A_{55}$ are defined in Equations (7) and (8).

### 2.2. Torsion of Orthotropic Plates with Transversal Shearing

**Figure 2:**
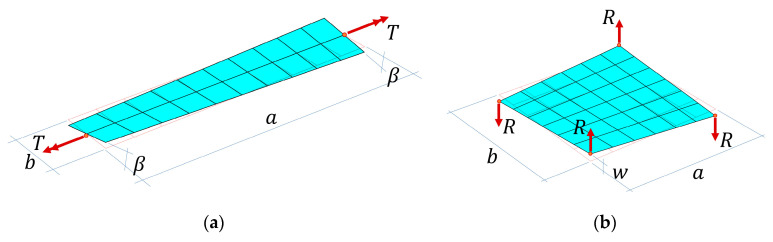
Two mechanical test setups to measure transversal shear stiffness of the sample: (**a**) via torque and angle of rotation and (**b**) via pair of force and vertical displacement (plate torsion test).

The foregoing constitutive equations incorporate a limiting-type orthotropy assumption due to sample size considered (a≫b, see Figure 2a), which causes $m_{yy}$ and $q_{y}$ to be reactive quantities, which enables a simple conversion from plate theory to a beam theory, in a physically justifiable way. This assumption leads to [25,26]:

(11) $$\frac{T}{\beta }=\frac{4D_{33}b}{a}\frac{1}{1+12\frac{D_{33}}{A_{44}}\frac{1}{b^{2}}}.$$

This formula represents the torsional stiffness of the sample including transversal shear stiffness along one of the direction, see Figure 2a, where T is the torque and β is the angle of rotation; a and b are dimensions of the sample.

Substituting the torque with a pair of forces, see Figure 2b, and modifying the above equation to incorporate both transversal shear stiffnesses, we received the following analytical form:

(12) $$\frac{R}{w}=\frac{16D_{33}b}{ab}\;\frac{1}{1+12D_{33}\left(\frac{1}{A_{55}}\frac{1}{a^{2}}+\frac{1}{A_{44}}\frac{1}{b^{2}}\right)}.$$

From hereon, this analytical approach will be abbreviated as AA. Here, R is the reaction force, while w is the vertical displacement. In the inverse form, the expression reads:

(13) $$\frac{w}{R}=\frac{ab}{16D_{33}}+\frac{3}{4}\left(\frac{1}{A_{55}}\frac{b}{a}+\frac{1}{A_{44}}\frac{a}{b}\right)$$

Determining the transversal shear stiffness of the sample from a static torsion plate test has been recently studied by the authors [3]. The study extended the work of Aviles et al. [2], where the additive form of w/R was used, in which nonphysical constant c was used. In the paper [3], constant c was replaced by the term $k_{1}k_{2}$, where $k_{1}$ and $k_{2}$ are described by material properties and sample geometry (this approach is abbreviated as AKK (approach $k_{1}\;k_{2}$) in forthcoming sections), so the final form of the expression reads:

(14) $$\frac{w}{R}=\frac{ab}{16D_{33}}+\frac{k_{1}k_{2}}{\sqrt{A_{44}A_{55}}},$$

where

(15) $$\begin{array}{ccc} k_{1}=\frac{2}{5}\left(\frac{b}{a}+\frac{7}{5}\right) & if & b>a,\\ k_{1}=\frac{2}{5}\left(\frac{a}{b}+\frac{7}{5}\right) & if & a\geq b, \end{array}$$

(16) $$\begin{array}{lll} k_{2}=\frac{3}{5}{\left(\frac{a}{b}\frac{G_{23}}{G_{13}}\right)}^{2/5} & if & G_{23}>G_{13},\\ k_{2}=\frac{3}{5}{\left(\frac{b}{a}\frac{G_{13}}{G_{23}}\right)}^{2/5} & if & G_{13}>G_{23},\\ k_{2}=1 & if & G_{13}=G_{23}. \end{array}$$

Further research showed that despite the fact that AKK [3] has good agreement with simple, but commonly used numerical solutions, it can be replaced with an exact solution AA, which is represented by Equation (13) or the numerical approach presented in one of the following subsections. The accuracies of the selected methods are checked on various examples in Section 3.

### 2.3. Reissner–Mindlin Composite Laminated Plate

Assuming the xyz coordinate system, the displacement field for composite laminated plates reads:

(17) $$u\left(x,y,z\right)=-z\theta_{x}\left(x,y\right),$$

(18) $$v\left(x,y,z\right)=-z\theta_{y}\left(x,y\right),$$

(19) $$w\left(x,y,z\right)=w_{0}\left(x,y\right),$$

where u, v and w are three unknown mid-surface displacements of the plate, while $\theta_{x}$ and $\theta_{y}$ are two rotations of the normal on the plane xz and yz from Equation (19):

(20) $$\theta_{x}=w_{,x}+\phi_{x};\;\theta_{y}=w_{,x}+\phi_{y},$$

noting that $\gamma_{x}=-\phi_{x}$ and $\gamma_{y}=-\phi_{y}$.

**Figure 3:**
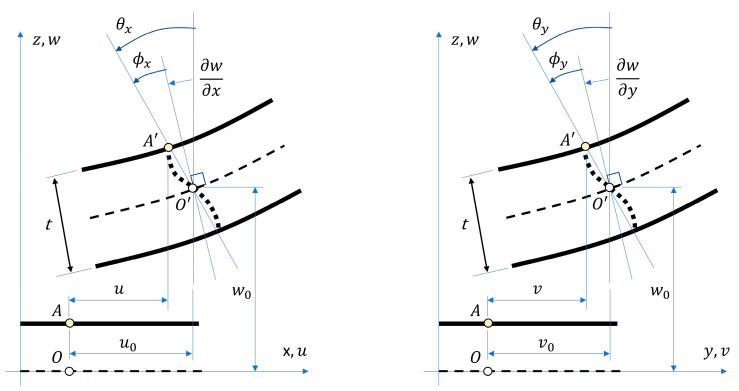
The displacements and the rotations of the normal-sign convention in Reissner–Mindlin plate theory.

In both the xz and yz vertical planes, the normal rotation was obtained as the sum of two rotations: (i) the corresponding slope of the middle plane of the plate and (ii) the additional rotation ϕ, which results from the lack of orthogonality of the normal to the middle plane after deformation, see Figure 3. Therefore, the rotations $\theta_{x}$ and $\theta_{y}$ cannot be calculated from the deflection only and become independent variables. This is the fundamental difference between Reissner–Mindlin and Kirchhoff–Love plate theories.

Relationships between strains (membrane—ε, bending—κ and shear—γ) and displacements read:

(21) ϵ=ε+zκ,

(22) $$\varepsilon =\left[\begin{array}{c} \varepsilon_{x}\\ \varepsilon_{y}\\ \gamma_{xy} \end{array}\right]=\left[\begin{array}{c} u_{,x}\\ v_{,y}\\ u_{,y}+v_{,x} \end{array}\right]=-z\left[\begin{array}{c} \theta_{x,x}\\ \theta_{y,y}\\ \theta_{x,y}+\theta_{y,x} \end{array}\right],$$

(23) $$\kappa =\left[\begin{array}{c} {𝜅}_{x}\\ {𝜅}_{y}\\ {𝜅}_{xy} \end{array}\right]=-\left[\begin{array}{c} \theta_{x,x}\\ \theta_{y,y}\\ \theta_{x,y}+\theta_{y,x} \end{array}\right]=-\left[\begin{array}{c} w_{,xx}\\ w_{,yy}\\ w_{,xy} \end{array}\right],$$

(24) $$\gamma =\left[\begin{array}{c} \gamma_{xz}\\ \gamma_{xy} \end{array}\right]=\left[\begin{array}{c} w_{,x}+u_{,z}\\ w_{,y}+v_{,z} \end{array}\right]=\left[\begin{array}{c} w_{,x}-\theta_{x}\\ w_{,y}-\theta_{y} \end{array}\right]=\left[\begin{array}{c} -\phi_{x}\\ -\phi_{y} \end{array}\right].$$

The stress–strain relations in local coordinates are given by:

(25) $$\left[\begin{array}{c} \sigma_{x}\\ \sigma_{y}\\ \sigma_{xy} \end{array}\right]=\left[\begin{array}{ccc} \frac{E_{11}}{1-\nu_{12}\nu_{21}} & \frac{\nu_{21}E_{11}}{1-\nu_{12}\nu_{21}} & 0\\ \frac{\nu_{12}E_{22}}{1-\nu_{12}\nu_{21}} & \frac{E_{22}}{1-\nu_{12}\nu_{21}} & 0\\ 0 & 0 & G_{12} \end{array}\right]\left[\begin{array}{c} \varepsilon_{x}\\ \varepsilon_{y}\\ \gamma_{xy} \end{array}\right],$$

(26) $$\left\{\begin{array}{c} \sigma_{xz}\\ \sigma_{yz} \end{array}\right\}=\frac{5}{6}\left[\begin{array}{cc} G_{13} & 0\\ 0 & G_{23} \end{array}\right]\left\{\begin{array}{c} \gamma_{xz}\\ \gamma_{yz} \end{array}\right\},$$

or in the compact form

(27) σ=C ϵ,

(28) $$\tau =C_{s}\;\gamma .$$

The strain energy, U, reads

(29) $$U=\frac{1}{2}\int_{A}\left(\epsilon^{T}D^{*}\epsilon +\gamma^{T}D_{s}^{*}\;\gamma \right)dA,$$

where

(30) $$D^{*}=\left[\begin{array}{cc} A & B\\ B & D \end{array}\right],$$

(31) $$D_{s}^{*}=D_{s}$$

and $A,B,D\;\mathrm{and}\;D_{s}$ are stiffnesses of the plates given by:

(32) $$\left(A,B,D\right)=\int_{-\frac{h}{2}}^{\frac{h}{2}}\left(1,z,z^{2}\right)C\left(z\right)dz,$$

(33) $$D_{s}=\int_{-h/2}^{h/2}C_{s}\left(z\right)dz.$$

**Figure 4:**
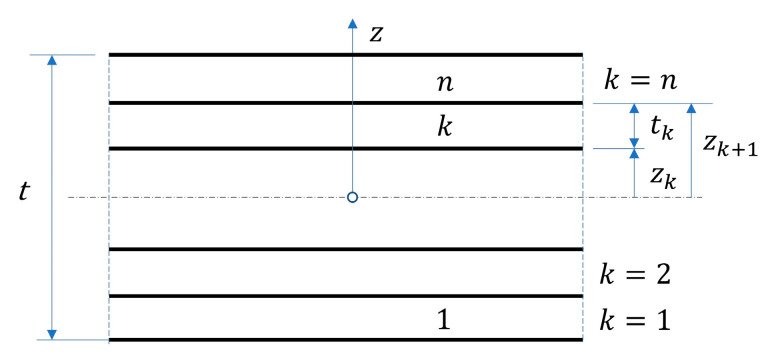
Laminate material of thickness t with n orthotropic homogeneous layers.

For an n-layer laminate with homogeneous orthotropic material within each of the k-th layers (Figure 4), Equations (32) and (33) can be rewritten

(34) $$A=\overset{n}{\underset{k=1}{\sum }}\left(z_{k+1}-z_{k}\right)C_{k},$$

(35) $$B=\overset{n}{\underset{k=1}{\sum }}\frac{1}{2}\left(z_{k+1}^{2}-z_{k}^{2}\right)C_{k},$$

(36) $$D=\overset{n}{\underset{k=1}{\sum }}\frac{1}{3}\left(z_{k+1}^{3}-z_{k}^{3}\right)C_{k},$$

(37) $$D_{s}=\overset{n}{\underset{k=1}{\sum }}\left(z_{k+1}-z_{k}\right)C_{sk},$$

where $C_{k}$ and $C_{sk}$. are the in-plane constitutive matrix, defined in Equations (25) and (26), for the *k*-th layer.

The matrix B becomes 0 if the laminate consists of homogeneous material, or the properties of a material are symmetrical with respect to the middle plane (z=0). The membrane and bending effects were then uncoupled and the neutral plane coincided with the plane xy. This means that the bending moments did not cause any membrane strains and the normal forces did not cause any curvature.

The work, V, done by the in plane and transverse load is given by:

(38) $$V=-\int_{A}q\;w\;dA.$$

The energy functional, Π, of the plate was finally obtained as follows:

(39) $$\Pi =U+V=\frac{1}{2}\int_{A}\left(\epsilon^{T}D^{*}\epsilon +\gamma^{T}D_{s}^{*}\;\gamma \right)dA-\int_{A}q\;w\;dA.$$

### 2.4. FEM Formulation of the Laminate Plate Element

For decades a finite element (FE) analysis has been a popular method for modeling advanced engineering problems. Due to its popularity and universality, the developers of FE software more and more often provide their users new functions, extending its capabilities. Following this trend, the software often allows one to include users’ material or element subroutines tailored for particular needs. Therefore, the FEM approach in comparison to the analytical approach is easier to be applied in the modern engineering tools. In the laminated plate element, the field variables, d, were approximated according to the associated node values as follows:

(40) $$d=\overset{N_{n}}{\underset{j=1}{\sum }}N_{j}\left(x,y\right)d_{j},$$

where $N_{n}$ represents number of nodes in the element; $N_{j}\left(x,y\right)$ is a shape function associated with node j;

(41) $$d_{j}^{T}=\left[\begin{array}{ccccc} u_{j} & v_{j} & w_{j} & \theta_{xj} & \theta_{yj} \end{array}\right]$$

is the displacement vector of the node degrees of freedom.

The membrane, bending and shear strains associated to the displacement in Equation (40) can be therefore obtained as follows:

(42) $$\varepsilon =\underset{j}{\sum }B_{j}^{m}d_{j},$$

(43) $$\kappa =\underset{j}{\sum }B_{j}^{b}d_{j},$$

(44) $$\gamma =\underset{j}{\sum }B_{j}^{s}d_{j},$$

where

(45) $$B_{j}^{m}=\left[\begin{array}{ccccc} N_{j,x} & 0 & 0 & 0 & 0\\ 0 & N_{j,y} & 0 & 0 & 0\\ N_{j,y} & N_{j,x} & 0 & 0 & 0 \end{array}\right],$$

(46) $$B_{j}^{b}=\left[\begin{array}{ccccc} 0 & 0 & 0 & N_{j,x} & 0\\ 0 & 0 & 0 & 0 & N_{j,y}\\ 0 & 0 & 0 & N_{j,y} & N_{j,x} \end{array}\right],$$

(47) $$B_{j}^{s}=\left[\begin{array}{ccccc} 0 & 0 & N_{j,x}\; & N_{j} & 0\\ 0 & 0 & N_{j,y} & 0 & N_{j} \end{array}\right].$$

Substituting Equations (40) and (42)–(44) into Equation (39) leads to:

(48) $$\Pi =\frac{1}{2}d\left[\int_{A}B^{T}D^{*}\;BdA+\int_{A}S^{T}D_{s}^{*}\;SdA\right]d-\left[\int_{A}qwdA\right]d,$$

where $B^{T}=\left[\begin{array}{cc} {\left(B^{m}\right)}^{T} & {\left(B^{b}\right)}^{T} \end{array}\right]$, $S^{T}={\left(B^{s}\right)}^{T}$. By using Lagrange’s equations for the energy expression in Equation (48), the characteristic equation of the system was obtained as follows:

(49) K d=P,

where

(50) $$K=\int_{A}\left(B^{T}D^{*}B+S^{T}D_{s}^{*}S\right)dA,$$

(51) $$P=\int_{A}q\;N\;dA.$$

It is noted that the shear locking phenomenon can appear in Equations (50) and (51) as the plate thickness decreased. To overcome this adverse the Reissner–Mindlin plate quadrilateral element with assumed transverse shear strain fields was adopted here. We used the plate element with 4 nodes with linear shear field, which initially was developed by Bathe and Dvorkin [27,28]. Its formulation bases on auxiliary transverse shear modes proposed by MacNeal [29,30] and Hughes et al. [31]. Later Donea and Lamain [32] and Onate et al. [33,34] derived the element using assumed strain concepts.

A standard 4-noded Q4 element [33,34] is characterized by a bilinear interpolation of deflections and rotations. The assumed transverse shear strain field is defined here in the natural system ξ,η as

(52) $$\gamma_{\xi }=\alpha_{1}+\alpha_{2}\eta ,$$

(53) $$\gamma_{\eta }=\alpha_{3}+\alpha_{4}\xi ,$$

(54) $$E=\left[\begin{array}{cccc} 1 & \eta & 0 & 0\\ 0 & 0 & 1 & \xi \end{array}\right].$$

**Figure 5:**
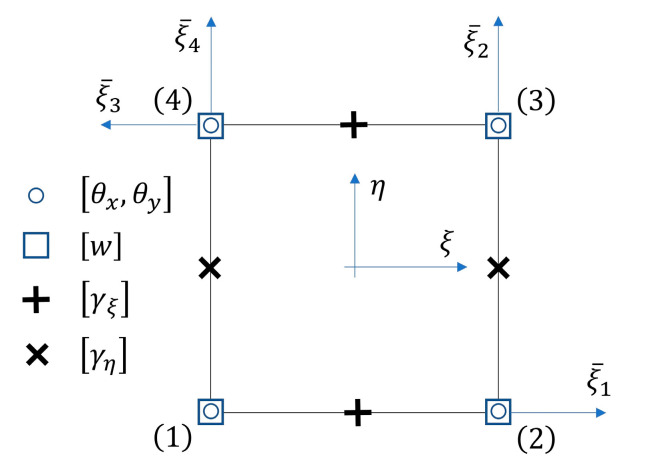
4-noded quadrilateral, bilinear deflection and rotations Reissner–Mindlin plate element with the assumed linear transverse shear strain field.

The $\alpha_{i}$ parameters can be found by taking the natural transverse shear strains $\gamma_{\bar{\xi }}$ at the all four middle-edge points shown in Figure 5, with

(55) $$\gamma_{\bar{\xi_{i}}}=\left(\alpha_{1}+\alpha_{2}\eta \right)\cos\beta_{i}+\left(\alpha_{3}+\alpha_{4}\xi \right)\sin\beta_{i};\;i=1,\ldots ,4,$$

where $\beta_{i}$ is the angle between the $\bar{\xi_{i}}$ and ξ axis. This leads to:

(56) $$\left\{\begin{array}{c} \alpha_{1}\\ \alpha_{2}\\ \alpha_{3}\\ \alpha_{4} \end{array}\right\}=P^{-1}\left\{\begin{array}{c} \gamma_{\bar{\xi }}^{1}\\ \gamma_{\bar{\xi }}^{2}\\ \gamma_{\bar{\xi }}^{3}\\ \gamma_{\bar{\xi }}^{4} \end{array}\right\},$$

where

(57) $$P=\left[\begin{array}{rrrr} 1 & -1 & 0 & 0\\ 0 & 0 & 1 & 1\\ -1 & -1 & 0 & 0\\ 0 & 0 & 1 & -1 \end{array}\right];\;P^{-1}=\frac{1}{2}\left[\begin{array}{rrrr} 1 & 0 & -1 & 0\\ -1 & 0 & -1 & 1\\ 0 & 1 & 0 & 1\\ 0 & 1 & 0 & -1 \end{array}\right].$$

The strains $\gamma_{\bar{\xi }}^{i}$ are related to $\gamma_{\xi }^{i}$, $\gamma_{\eta }^{i}$ by

(58) $$\left\{\begin{array}{c} \gamma_{\bar{\xi }}^{1}\\ \gamma_{\bar{\xi }}^{2}\\ \gamma_{\bar{\xi }}^{3}\\ \gamma_{\bar{\xi }}^{4} \end{array}\right\}=\left[\begin{array}{cccccccc} 1 & 0 & 0 & 0 & 0 & 0 & 0 & 0\\ 0 & 0 & 0 & 1 & 0 & 0 & 0 & 0\\ 0 & 0 & 0 & 0 & -1 & 0 & 0 & 0\\ 0 & 0 & 0 & 0 & 0 & 0 & 0 & 1 \end{array}\right]\left\{\begin{array}{c} \gamma_{\xi }^{1}\\ \gamma_{\eta }^{1}\\ \vdots \\ \gamma_{\xi }^{4}\\ \gamma_{\eta }^{4} \end{array}\right\}=T\;{\hat{\gamma }}^{\prime }.$$

The relationship between the Cartesian transverse shear strains at the middle-edge points and the natural transverse shear strains is:

(59) $${\hat{\gamma }}^{\prime }=\left[\begin{array}{cccc} J^{1} & 0 & 0 & 0\\ 0 & J^{2} & 0 & 0\\ 0 & 0 & J^{3} & 0\\ 0 & 0 & 0 & J^{4} \end{array}\right]\left\{\begin{array}{c} {\hat{\gamma }}^{1}\\ \vdots \\ {\hat{\gamma }}^{4} \end{array}\right\}=Z\;\hat{\gamma };\;{\hat{\gamma }}^{i}=\left\{\begin{array}{c} \gamma_{xz}^{i}\\ \gamma_{yz}^{i} \end{array}\right\}.$$

The Cartesian transverse shear strains at the four sampling points are then related to the nodal displacements by:

(60) $$\hat{\gamma }=\left\{\begin{array}{c} B_{s}^{1}\\ B_{s}^{2}\\ B_{s}^{3}\\ B_{s}^{4} \end{array}\right\}a^{\left(e\right)}=B_{s}\;a^{\left(e\right)}.$$

The substitute transverse shear strain matrix was obtained by

(61) $${\bar{B}}_{s}=J^{-1}\;E\;P^{-1}\;T\;Z\;{\hat{B}}_{s}.$$

Here, the element is described as QLLL (for quadrilateral, bilinear deflection and rotations and linear transverse shear strain fields). The element satisfies conditions [35,36,37]

(62) $$n_{\theta }+n_{w}\geq n_{\gamma },$$

(63) $$n_{\gamma }\geq n_{w},$$

where $n_{\theta }$, $n_{w}$ and $n_{\gamma }$ are the number of variables included in the interpolation of the rotations, the deflection and the transverse shear strains, respectively. In order to preserve the element from spurious mechanisms the full 2 × 2 quadrature for all terms were used in computation of the stiffness matrix. Since the shear forces and bending moments are constant along each natural direction, the fine meshes are required for certain applications.

The product $AP^{-1}T$ in Equation (61) is

(64) $$E\;P^{-1}\;T=\frac{1}{2}\left[\begin{array}{ccccccccc} 1-\eta & 0 & 0 & 0 & | & 1+\eta & 0 & 0 & 0\\ 0 & 0 & 0 & 1+\xi & | & 0 & 0 & 0 & 1-\xi \end{array}\right],$$

The assumed transverse shear strain field can be also written in the direct form:

(65) $$\gamma_{\xi }=\frac{1}{2}\left(1-\eta \right)\gamma_{\xi }^{1}+\frac{1}{2}\left(1+\eta \right)\gamma_{\xi }^{3},$$

(66) $$\gamma_{\eta }=\frac{1}{2}\left(1+\xi \right)\gamma_{\eta }^{2}+\frac{1}{2}\left(1-\xi \right)\gamma_{\eta }^{4}.$$

## 3. Results

The efficiency of the analytical approach AA presented in the paper was verified in reference to several models using different methods, both numerical and analytical. Results from the numerical methods were obtained from the commercial FE software, namely Abaqus FEA, which enables a few types of FEs to model plates. Results from the AKK method [3] was used in the comparison as the example of the quasi-analytical method, see Equation (14).

To verify the method in a broad application, key geometrical and material parameters of the samples were changed in a wide range. The following parameters were analyzed: the aspect ratio of the sample and the shear stiffnesses, i.e., $D_{33}$, $A_{44}$ and $A_{55}$. On the basis of preliminary tests, it was concluded that two sizes of samples should be analyzed for corrugated materials, in which a×b equals, e.g., 75 mm×75 mm and 125 mm×25 mm; the aspect ratios equal 1:1 and 5:1, respectively. Regarding torsion stiffness, $D_{33}$ had two values, namely, $D_{33}=450$ Nmm and $D_{33}=900$ Nmm. The transversal shear stiffness, $A_{44}$ and $A_{55}$, varied from 5 to 250 N/mm, with a few selected values in between.

**Figure 6:**
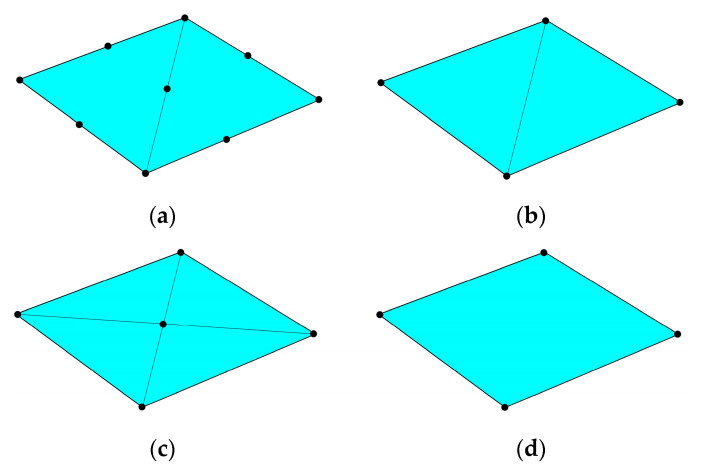
Meshes of finite element models used in the study: (**a**) STRI65, (**b**) S32, (**c**) S34 and (**d**) S4/S4R.

**Figure 7:**
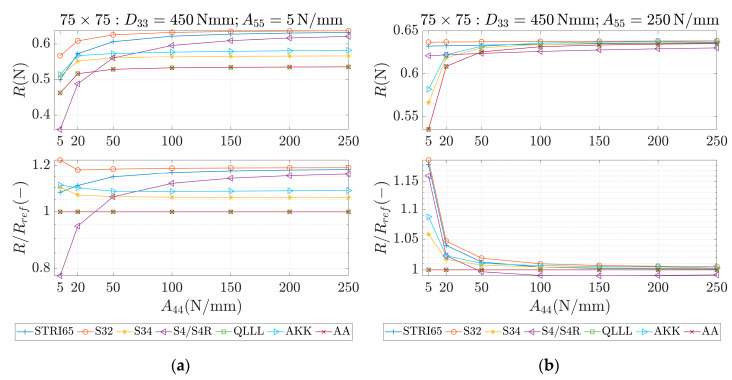
Reactions (first row) and reactions to the reference (analytical) solution (second row) for 75 × 75 sample, $D_{33}=450$ Nmm and different values of $A_{44}$. (**a**) $A_{55}=5$ N/mm and (**b**) $A_{55}=250$ N/mm.

**Figure 8:**
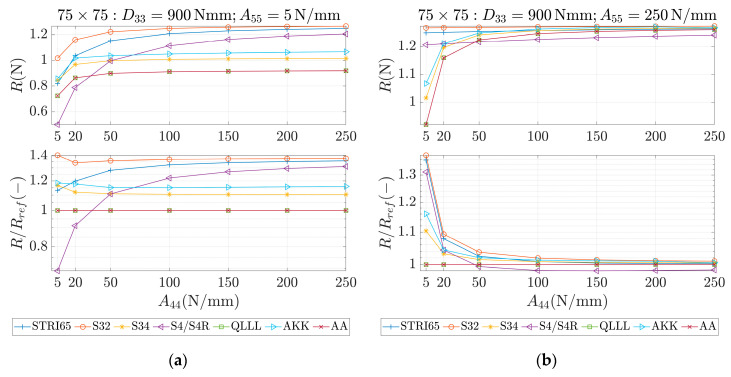
Reactions (first row) and reactions to the reference (analytical) solution (second row) for 75 × 75 sample, $D_{33}=900$ Nmm and different values of $A_{44}$. (**a**) $A_{55}=5$ N/mm and (**b**) $A_{55}=250$ N/mm.

**Figure 9:**
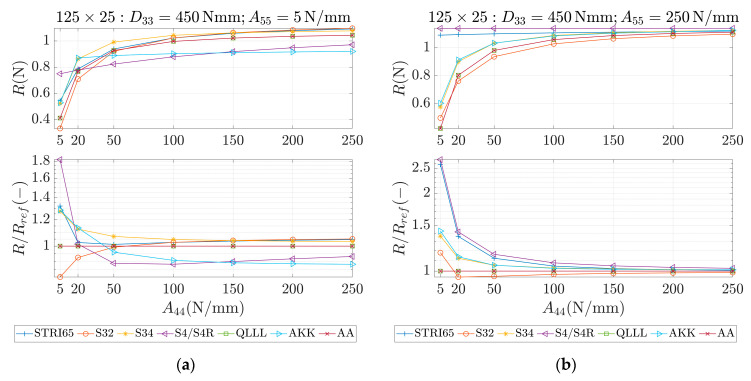
Reactions (first row) and reactions to the reference (analytical) solution (second row) for 125 × 25 sample, $D_{33}=450$ Nmm and different values of $A_{44}$. (**a**) $A_{55}=5$ N/mm and (**b**) $A_{55}=250$ N/mm.

**Figure 10:**
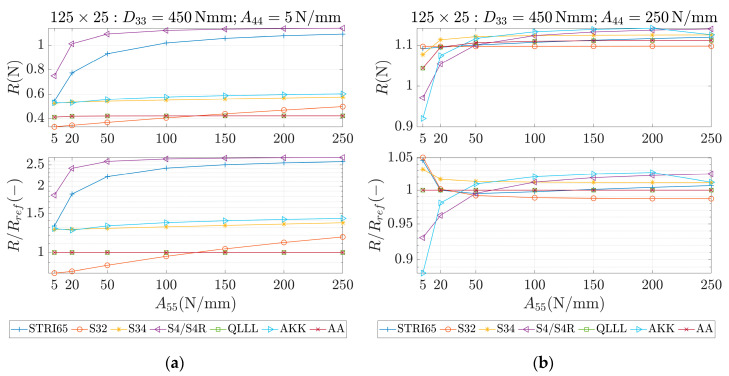
Reactions (first row) and reactions to the reference (analytical) solution (second row) for 125 × 25 sample, $D_{33}=450$ Nmm and different values of $A_{55}$. (**a**) $A_{44}=5$ N/mm and (**b**) $A_{44}=250$ N/mm.

**Figure 11:**
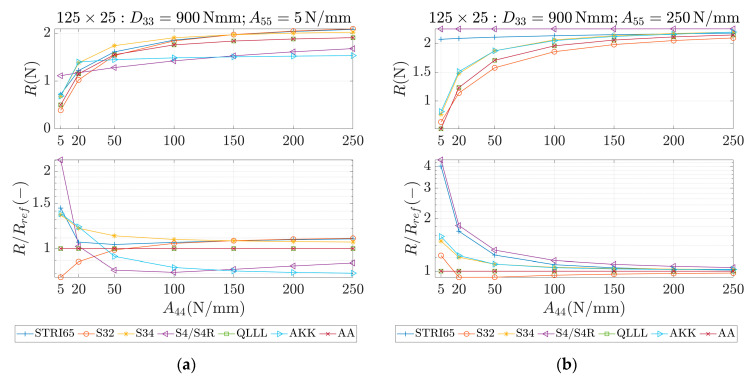
Reactions (first row) and reactions to the reference (analytical) solution (second row) for 125 × 25 sample, $D_{33}=900$ Nmm and different values of $A_{44}$. (**a**) $A_{55}=5$ N/mm and (**b**) $A_{55}=250$ N/mm.

**Figure 12:**
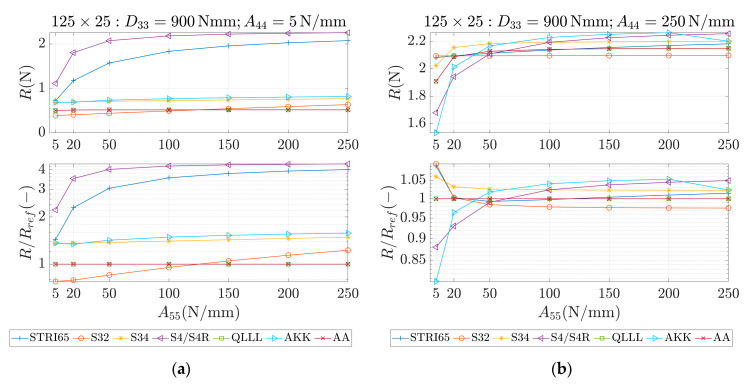
Reactions (first row) and reactions to the reference (analytical) solution (second row) for 125 × 25 sample, $D_{33}=900$ Nmm and different values of $A_{55}$. (**a**) $A_{44}=5$ N/mm and (**b**) $A_{44}=250$ N/mm.

These parameters were used as an input for different methods, which are marked in the study as STRI65, S32, S34, S4/S4R, QLLL, AA and AKK. In all of those methods, the force was applied in the corners, like in Figure 2b. STRI65 refers to the FE model, in which two 6-node triangular thin shell FEs, using five degrees of freedom per node, were used, see Figure 6a. S32 refers to the FE model, in which two 3-node triangular general-purpose shell FEs were used, see Figure 6b. S34 refers to the FE model, in which four 3-node triangular general-purpose shell FEs were used, see Figure 6c. S4/S4R refers to the FE models, in which one 4-node general-purpose shell FE was used with full and reduced integration scheme, respectively, see Figure 6d. QLLL refers to the FE model, in which 4-node shell FE presented in [33,34] was used, see Equations (52)–(66) and Figure 5. AA refers to the analytical approach presented in this paper, see Equation (12), while AKK refers to the analytical approach presented in the paper of Garbowski, Gajewski and Grabski [3]. The results obtained from application all these methods are shown in Figure 7, Figure 8, Figure 9, Figure 10, Figure 11 and Figure 12. All data used for generation the plots are included in Supplementary Materials.

In the first row of each graph (Figure 7, Figure 8, Figure 9, Figure 10, Figure 11 and Figure 12), the reaction forces, R, are presented for particular parameters of a×b, $D_{33}$, $A_{44}$ and $A_{55}$, while in the second row these reaction forces in reference to the analytical solutions of the AA model, see Equation (12), $R/R_{ref}$, are shown. In Figure 7 and Figure 8, square samples of 75 mm×75 mm were assumed, while in Figure 9, Figure 10, Figure 11 and Figure 12 the samples were rectangular—125 mm×25 mm. In Figure 7, Figure 9 and Figure 10, the assumed value of $D_{33}$ equaled 450 Nmm while in Figure 8, Figure 11 and Figure 12, $D_{33}$ equaled 900 Nmm. On the horizontal axis, the variability of $A_{44}$ is shown in Figure 7, Figure 8 and Figure 9 and Figure 11, while the variability of $A_{55}$ is shown in Figure 10 and Figure 12.

## 4. Discussion

The results demonstrated in Section 3 applied to a variety of cardboard structures, which is reflected in a wide shear stiffness represented by the output reaction forces. In this study, the reaction forces were in the range from 0.5 to 2.5 N. Such comprehensive verification allows several useful conclusions to be drawn for a more robust modeling of the corrugated cardboard structures. First, it should be noted that the analysis with the QLLL element produced results that were perfectly consistent with the analytical solution AA, see Figure 7, Figure 8, Figure 9, Figure 10, Figure 11 and Figure 12. This fact was observed in each of the 196 analyzed cases. S34 method was usually the second-best FE method under consideration, comparing its accuracy with respect to the reference analytical solution, for instance see Figure 7a or Figure 12b. This was not always obvious, e.g., see Figure 11a, or true, e.g., see Figure 9b or Figure 10a.

In the results, there were crucial differences while comparing rectangular (125 mm×25 mm) and square (75 mm×75 mm) samples. The biggest divergence from the analytical solution were observed in STRI65 and S4/S4R methods for rectangular sample, especially in cases, when the sample had a large transversal shear stiffness along short dimension and small transversal shear stiffness along long dimension, see Figure 11b ($A_{44}=5$ N/mm), and Figure 12a ($A_{55}>50$ N/mm). In these cases, the difference in relation to the reference solution was even four times greater. Moreover, for these cases, the reaction force was relatively low, i.e., about 0.5 N. The STRI65 and S4/S4R approached greatly overestimated transversal shear stiffness of the samples.

On contrary, also in rectangular samples, if the material had large transversal shear stiffness along longer dimension of the sample, the differences between all methods and the reference solution were very low, see Figure 10b and Figure 12b. In all these methods, apart from the S34 case, the reactions changed their relation to the reference solution by crossing the value of 1.0. In other words, the analyzed methods for some values of $A_{55}$ overestimated the analytical solution and for other values of $A_{55}$, underestimated. Going further, while considering AKK in the rectangular samples; the method in 50% of cases may be considered as equally good comparing to the S34 method, but both methods were worse than S32. In the other 50% of cases, AKK was worse than S32 and S34, which were in these cases equally good methods. In all cases, AKK was more accurate than STRI65 and S4/S4R.

In the results for square samples, over an entire range of transversal shear stiffness considered, AKK was competitive to the S34 method, but usually slightly worse. In these cases, the S32 method was usually much worse than AKK or S34. This was shown, for example, in Figure 8a.

It is worth noting that for the square sample, due to the symmetry of the material ($A_{44}$ vs. $A_{55}$), and thus the symmetry of the results, these plots were not presented to avoid repetition.

One of the limitations of the study was considering the finite ranges of the material parameters of $D_{33}$, $A_{44}$ and $A_{55}$. The ranges adopted here, even if they did not appear to be physically reasonable for the typical properties of corrugated board, may be of interest when analyzing sandwich panels made of other materials. Another limitation was fixing the two sets of in-plane dimensions and only one thickness of the samples. The selection of the samples dimensions was followed by a practical laboratory tests of corrugated board [38], while the selected thickness is one of the most commonly used in the corrugated cardboards packaging industry.

## 5. Conclusions

In the paper, the classic governing equations of the Reissner–Mindlin plate were presented with a particular application to composite laminates. The formula for torsion of the orthotropic plate including transversal shear stiffness was defined and compared with the recent considerations from the literature. Transverse shear is an important component if the analyzed plate is thick with respect to the in-plane dimensions and/or its core has significantly lower stiffness than the outer faces. Different methods for modeling such a plate, both numerical and analytical, were selected to compute their accuracies comparing to the exact, analytical solution. Numerical methods used varied in the type of finite element used and number of elements taken into consideration. To cover a wide range of material properties, the computations were performed for selected in-plane and transversal shear moduli. Samples with two different aspect ratios, rectangular and square, were tested.

It was concluded that the 4-noded Reissner–Mindlin plate quadrilateral with assumed linear transverse shear strain field gave the best performance. Results obtained via this method were exact with the analytical solution. The results from other methods, with 6-node triangular elements or 4-node quadrilateral elements (both, with full integration or reduced), with commonly used finite elements were essentially worse. In the worst cases, the overestimation of the force was even up to four times. In this comparison, the 3-node triangular element performance was surprisingly moderately good. Moreover, the differences between rectangular and square samples were significant. It should be noted that in rectangular samples, if the material had a large transversal shear stiffness along a longer dimension of the sample, the differences of all methods to the reference solution were very low.

## Supplementary Materials

The following are available online at https://www.mdpi.com/1996-1944/13/21/5016/s1. All computational results are presented in CSV files, including the data, based on which Figure 7, Figure 8, Figure 9, Figure 10, Figure 11 and Figure 12 were generated. In these files, 1st and 2nd columns are a and b dimensions of the box, respectively, 3rd column is the thickness of the wall, the materials parameters: $G_{12}$, $G_{13}$, $G_{23}$, $D_{33}$, $A_{44}$ and $A_{55}$. are given in the columns 4th to 9th, respectively. The columns 10th–16th include results of the calculations using: STRI65, S32, S34, S4/S4R, QLL, AKK and AA, respectively.
